# A Case of Differential Lung Ventilation Using a Double-Lumen Endotracheal Tube via Tracheostomy in a Patient With Acquired Laryngeal Web Undergoing Lobectomy

**DOI:** 10.7759/cureus.82390

**Published:** 2025-04-16

**Authors:** Yuki Yamaguchi, Takashi Matsusaki, Daisuke Ono, Ryuji Kaku

**Affiliations:** 1 Department of Anaesthesiology, Mie University Hospital, Tsu, JPN

**Keywords:** acquired laryngeal web, difficult airway management, double-lumen endotracheal tube for tracheostomy, pulmonary lobectomy, tracheostomy

## Abstract

Acquired laryngeal web is associated with difficult airway management during anesthesia induction or extubation. We experienced a case of lung resection under general anesthesia in a patient who was preoperatively diagnosed with a laryngeal web. The patient was a 78-year-old female (height: 153 cm, weight: 59 kg, BMI: 25.1 kg/m²) scheduled for right lung lobe resection under general anesthesia. She had been diagnosed with a laryngeal web fourteen years earlier and had undergone surgery twice to improve the airway stricture. Since preoperative laryngoscopic assessment indicated the potential for laryngeal edema due to stimulation from tracheal intubation and extubation, we decided to perform a tracheostomy under regional anesthesia before the induction of general anesthesia. After securing a reliable airway, we induced general anesthesia and replaced the tracheostomy tube with a left-sided double-lumen tube via the tracheostomy. We were able to successfully conduct stable one-lung ventilation without any intraoperative complications. Her postoperative course was relatively stable, and the tracheal tube was removed on postoperative day two. She has subsequently not experienced any stricture-related symptoms.

It is important to reliably secure the airway via a surgical approach instead of tracheal intubation in adult patients with an acquired laryngeal web, as laryngeal stimulation can easily cause edema. We considered a surgical airway to be essential in this patient with an acquired laryngeal web undergoing surgery under general anesthesia.

## Introduction

Laryngeal web is a rare congenital defect that can also be acquired. It is a condition where a membrane tissue forms in the larynx [1]. The first case of a laryngeal web was described by Fleischmann in 1820 after performing an autopsy on a 27-day-old infant. In 1869, Zurhelle described the first living case, diagnosed by indirect laryngoscopy, in an 11-year-old boy who presented with a voice anomaly. Congenital laryngeal web constitutes 5% of all congenital laryngeal anomalies. Most congenital webs present either at birth or in the first few months of life. Very rarely, the web may present itself even in an older age group.

On the contrary, the primary cause of acquired laryngeal web is often unknown but could result from long-term intubation [2,3]. While both forms are rare, there are reports regarding difficult airway management in patients with laryngeal web [4,5]. The fundamental treatment for laryngeal web is direct surgical release of the stenotic site; however, postoperative re-stenosis of the glottis is problematic, often requiring invasive surgical tracheostomy. It was challenging to select intraoperative airway management for the patient with acquired laryngeal web because we did not have any similar experience. Therefore, we chose tracheostomy in advance to proceed with surgery. However, the anesthesia management for lung resection was also challenging, especially in the patient with tracheostomy, because one-lung ventilation was necessary. In order to secure one-lung ventilation, we chose to use a double-lumen endotracheal tube for tracheostomy [6]. We managed a case of acquired laryngeal web using a double-lumen endotracheal tube through the tracheostomy opening, successfully facilitating the airway management for lobectomy.

This article was previously presented as a meeting abstract at the 2024 IARS Annual Meeting on May 19, 2024.

## Case presentation

**Figure 1:**
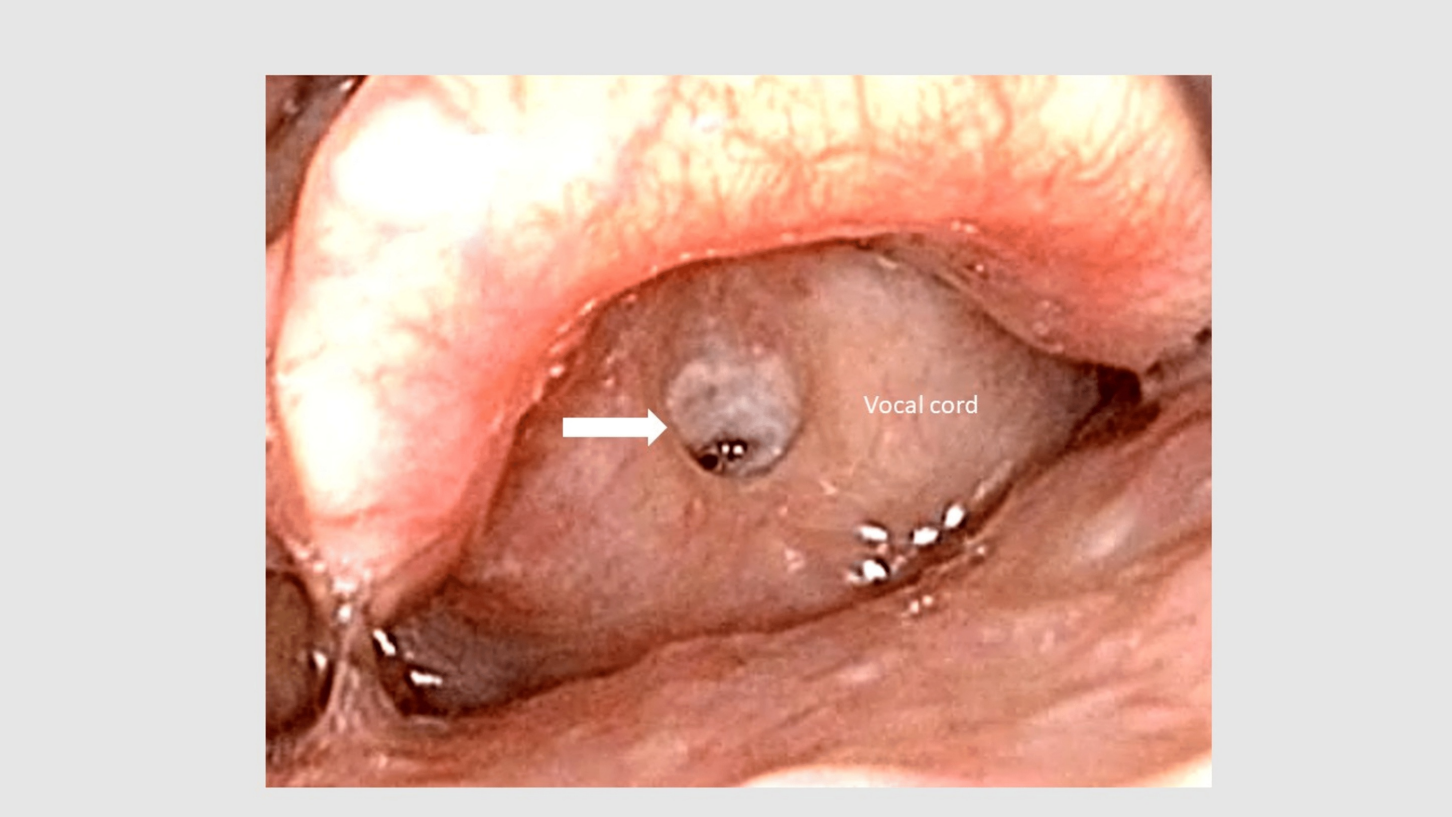
Larynx as seen by laryngoscopy. Preoperative stenosis of the glottis. The patient’s larynx appeared narrowed due to previous surgery for an acquired laryngeal web, as seen by laryngoscopy and indicated by the black arrow. Permission for publication was obtained from the patient.

A 78-year-old female (BMI: 25.1 kg/m²) presented with dyspnea due to a laryngeal web detected fourteen years ago. At that time, two separate operations were required to directly release the stenotic site. She did not have any difficulty breathing postoperatively, but a tendency for re-stenosis of the larynx was diagnosed. Lung cancer in her right upper lobe was incidentally detected during a subsequent medical examination, and she was scheduled for surgery. Given the predicted difficulty with airway management perioperatively, she was admitted to our academic hospital. She did not exhibit any upper airway symptoms preoperatively. Her respiratory examination showed a slight decrease in function (vital capacity (VC): 2.6 liters, percent vital capacity (%VC): 91%; forced expiratory volume in 1 second (FEV1): 1.5 liters, FEV1%: 74.9%). Cervical CT showed the narrowest diameter of the trachea was 4.99 mm × 9.58 mm, and laryngeal endoscopy showed stenosis of the glottis compared to the previous one (Figure 1). The grade of the laryngeal web was Ⅳ. We discussed whether we should conduct a usual rapid induction of anesthesia (oral intubation approach) with the surgeons, but finally decided on tracheostomy under light sedation prior to lung surgery.

**Figure 2:**
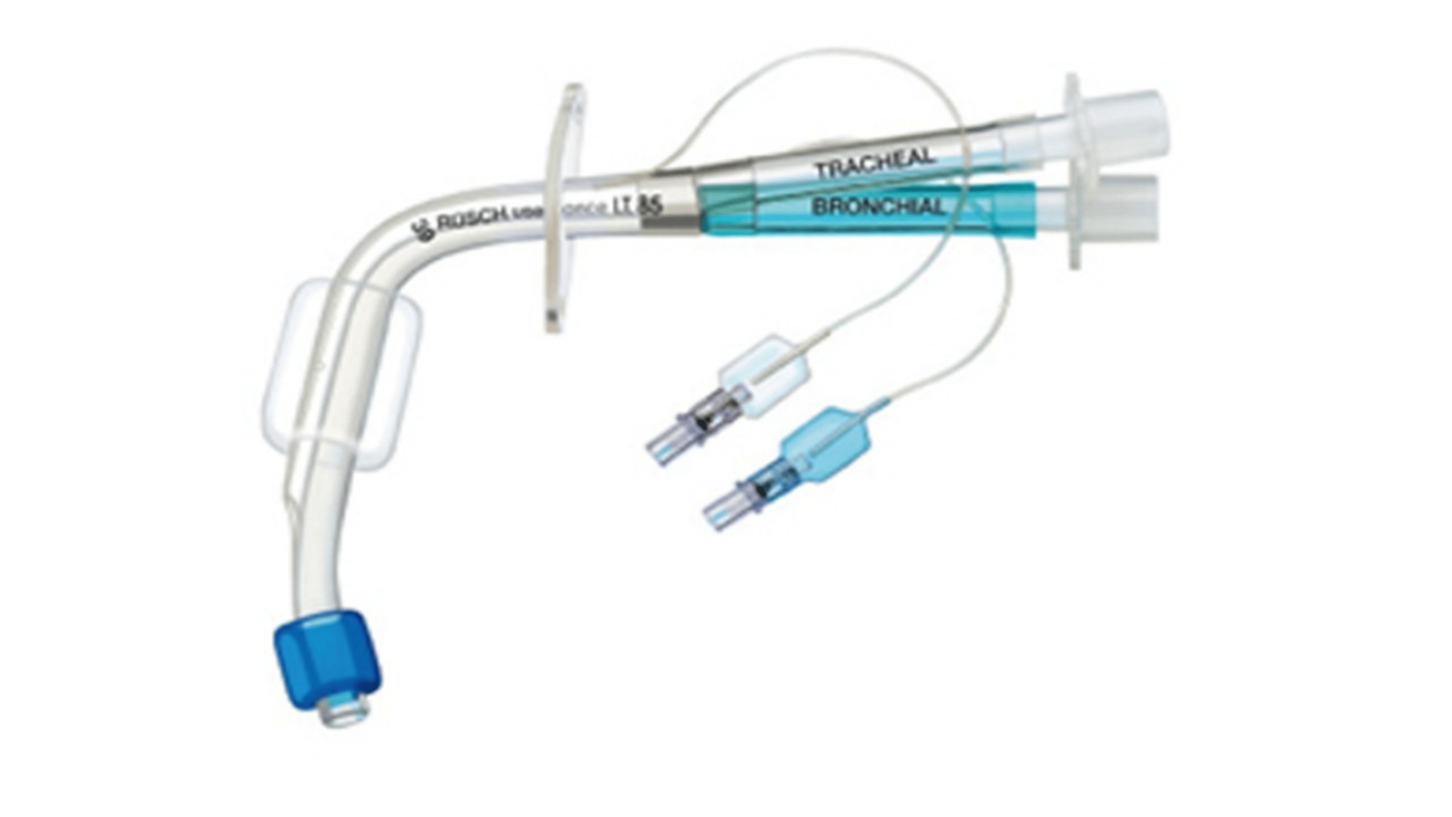
Photograph of double-lumen endotracheal tube for tracheostomy. RUSH Tracheopart, 75 mm, 39 Fr, inner diameter (ID): 13.9 mm.

**Figure 3:**
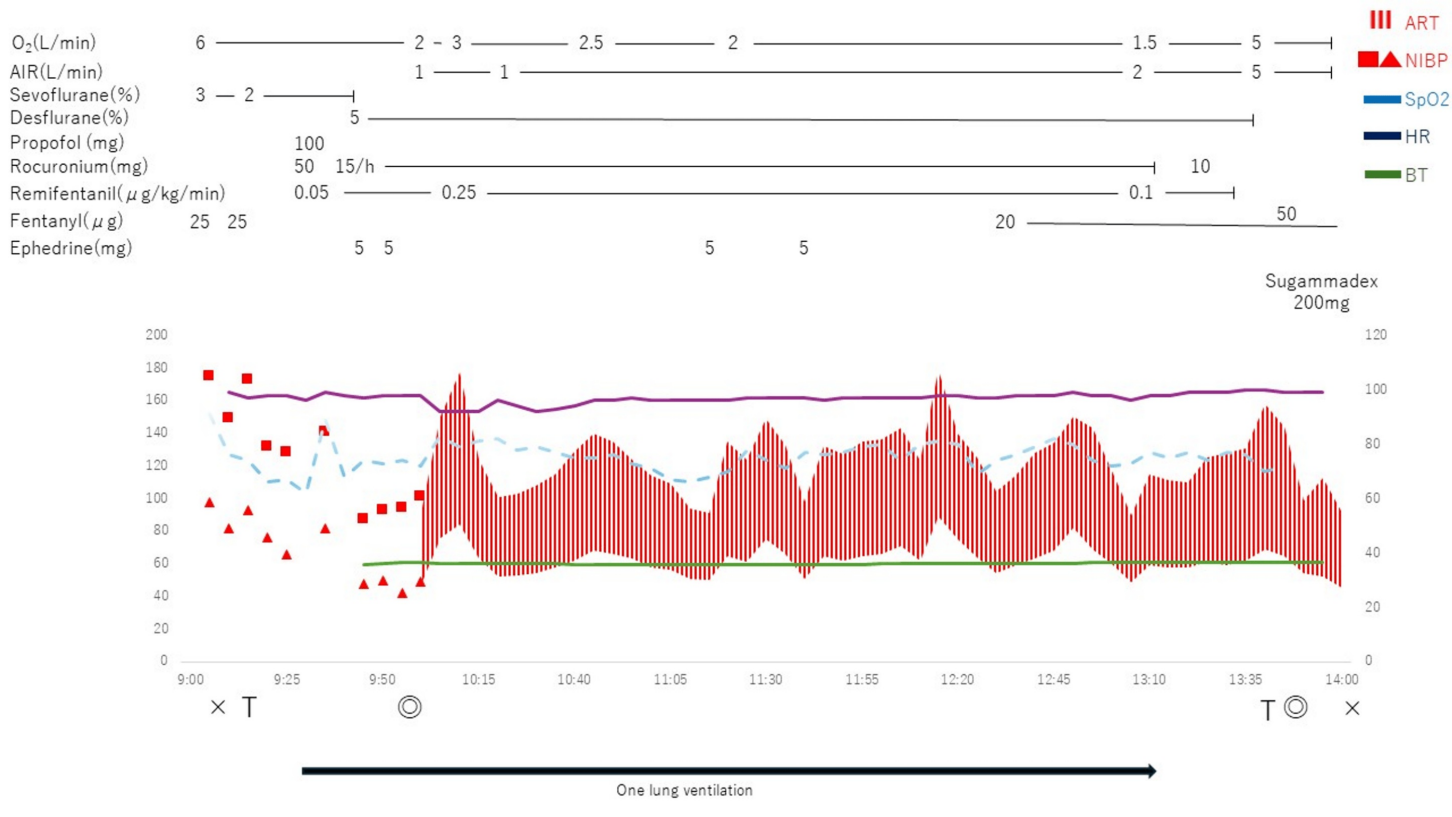
Anesthesia record.

We conducted the tracheostomy using fentanyl and sevoflurane with regional anesthesia (1% lidocaine with epinephrine), and then introduced general anesthesia using neuromuscular blocking agents. We changed the regular tracheostomy tube to a double-lumen endotracheal tube for tracheostomy (RUSH tracheopart 75 mm, 39 Fr, ID 13.9 mm) smoothly without any complications (Figure 2). We confirmed the correct position of the tube’s tip by performing fibre-optic bronchoscopy. General anesthesia was maintained using air-oxygen-sevoflurane and remifentanil. The patient was kept in the left lateral position for the endoscopic right upper lobectomy. Although there was a temporary desaturation event during one-lung ventilation, increasing the positive end-expiratory pressure (PEEP) from 5 to 10 cm H₂O quickly stabilized the patient's respiratory condition. We did not encounter any problems regarding one-lung ventilation during surgery. Finally, the lobectomy (adenocarcinoma) was also completed successfully without any further perioperative complications. The patient was returned to the supine position, and the double-lumen endotracheal tube was switched back to a regular tracheostomy tube (8.5 Fr) (Figure 3). Her postoperative course was uneventful, and she was discharged from the ICU to the general ward on postoperative day one. Regular follow-up with an otolaryngologist confirmed no re-stenosis of her larynx. The tracheostomy was closed on postoperative day 15, and since her swallowing function remained normal, she was discharged on postoperative day 17.

## Discussion

Laryngeal web is a condition in which membranous tissue forms in the larynx, either congenitally or in an acquired manner. The main cause of congenital laryngeal web is an abnormality in laryngeal development during the fetal period, while the primary cause of acquired laryngeal web is scarring or granuloma formation due to inflammation or trauma to the larynx. Previous reports have shown that the diagnosis of laryngeal web was often made during unexpected intubation, and there are reports that long-term intubation can cause acquired laryngeal web [7,8,9].

There have been several reported cases concerning intraoperative anesthesia management for patients with laryngeal web. Singh PM and Khanna P reported a case of refractory ventilation due to intraoperative bronchospasm, which was later diagnosed as laryngeal web by postoperative laryngoscopy [9]. Schmidt MH et al., in a case similar to ours, attempted to use a double-lumen tube for lung resection but found it too difficult to insert due to the presence of a laryngeal web [10]. Basaranoglu G et al. also reported a case of difficult endotracheal intubation with Ralinke edema caused by a laryngeal web. Most of these cases were diagnosed after encountering difficulty with airway management. In our case, the laryngeal web was fortunately diagnosed before surgery, allowing us to plan thoroughly to secure the airway. Laryngeal webs are classified into four grades based on the degree of laryngeal stenosis according to Cohen’s classification. Our case was Type 3, involving 50% to 75% of the glottis, according to Cohen’s classification. The structure of the larynx in our case was thicker anteriorly and thinner posteriorly. Based on Benjamin’s classification, our case was classified as a glottic web. Fortunately, our patient also agreed to our recommendation to proceed with awake tracheostomy.

The definite cause and mechanism of laryngeal web are still not fully clarified; however, surgery is often necessary as a radical treatment, depending on the patient's dyspnea or symptoms of airway obstruction. In our case, previous surgery had been conducted, but the airway stricture gradually progressed without any noticeable respiratory symptoms. If the procedure to address airway stenosis were delayed, the patient might have died due to difficulty in ventilation. As in our case, emergent tracheostomy would be necessary. Our case involved a patient with a history of surgery for laryngeal stenosis, who still had a narrowed larynx compared to a healthy person but exhibited no subjective symptoms. For the lung resection, we considered whether to use a double-lumen tube orally, which could have worsened the laryngeal web or caused laryngeal edema due to trauma to the larynx. We ultimately decided to secure the airway by performing a surgical tracheostomy before inducing anesthesia and inserting the double-lumen tube for tracheostomy. We also considered the option of using usual oral intubation with a bronchial blocker, but prioritized safety and chose surgical tracheostomy before anesthesia induction. This decision was based on laryngoscopic findings and discussion with the otolaryngologist. Each case should be considered individually based on the degree of stricture and clinical circumstances when selecting the airway method. There have even been successful cases of airway management in laryngeal web using a laryngeal mask [11].

It was challenging to perform one-lung ventilation through the tracheostomy opening. For lung resection, our usual practice involves oral intubation using a double-lumen tube or a bronchial blocker, if possible. However, we could not attempt oral intubation due to the laryngeal stricture. The next plan was to insert a thicker tracheostomy tube or spiral tube with a bronchial blocker; however, tube fixation was difficult because of the lateral position. Therefore, we decided to use a double-lumen tube for tracheostomy. It was easy to manage one-lung ventilation using the RUSCH Tracheopart 116400-75R left-sided tube (Toray, Japan).

## Conclusions

We successfully achieved differential lung ventilation using a double-lumen endotracheal tube for tracheostomy in a patient with an acquired laryngeal web. We considered whether to attempt awake intubation or to perform the tracheostomy under light sedation; however, we ultimately decided on tracheostomy first based on observations from laryngoscopy. The preoperative tracheostomy facilitated perioperative management during lobectomy in this case without any complications. The double-lumen endotracheal tube for tracheostomy proved to be more useful and convenient for securing one-lung ventilation than we had expected. Airway management in a patient with an acquired laryngeal web requires careful consideration of the surgical procedure, the patient's characteristics, and the condition of the larynx.
